# A General Theoretical Framework to Study the Influence of Electrical Fields on Mesenchymal Stem Cells

**DOI:** 10.3389/fbioe.2020.557447

**Published:** 2020-10-20

**Authors:** Jonathan Dawson, Poh Soo Lee, Ursula van Rienen, Revathi Appali

**Affiliations:** ^1^Department of Computer Science and Electrical Engineering, Institute of General Electrical Engineering, University of Rostock, Rostock, Germany; ^2^Max Bergmann Center for Biomaterials, Institute for Materials Science, Technical University of Dresden, Dresden, Germany; ^3^Department of Ageing of Individuals and Society, Interdisciplinary Faculty, University of Rostock, Rostock, Germany; ^4^Department of Life, Light and Matter, Interdisciplinary Faculty, University of Rostock, Rostock, Germany

**Keywords:** mathematical modeling, mean-field approach, data-driven modeling, stem cell differentiation, electrical stimulation, human mesenchymal cells

## Abstract

Mesenchymal stem cell dynamics involve cell proliferation and cell differentiation into cells of distinct functional type, such as osteoblasts, adipocytes, or chondrocytes. Electrically active implants influence these dynamics for the regeneration of the cells in damaged tissues. How applied electric field influences processes of individual stem cells is a problem mostly unaddressed. The mathematical approaches to study stem cell dynamics have focused on the stem cell population as a whole, without resolving individual cells and intracellular processes. In this paper, we present a theoretical framework to describe the dynamics of a population of stem cells, taking into account the processes of the individual cells. We study the influence of the applied electric field on the cellular processes. We test our mean-field theory with the experiments from the literature, involving *in vitro* electrical stimulation of stem cells. We show that a simple model can quantitatively describe the experimentally observed time-course behavior of the total number of cells and the total alkaline phosphate activity in a population of mesenchymal stem cells. Our results show that the stem cell differentiation rate is dependent on the applied electrical field, confirming published experimental findings. Moreover, our analysis supports the cell density-dependent proliferation rate. Since the experimental results are averaged over many cells, our theoretical framework presents a robust and sensitive method for determining the effect of applied electric fields at the scale of the individual cell. These results indicate that the electric field stimulation may be effective in promoting bone regeneration by accelerating osteogenic differentiation.

## 1. Introduction

Human mesenchymal stem cells (hMSCs) possess a unique capability of self-renewal and differentiation into cells of various types of tissues, such as bone, cartilage, and adipose. Thus, the hMSCs are the promising cell types for regenerative medicine and tissue engineering. The gene expression levels of an hMSC are known to be the decisive regulators of hMSCs differentiation. These gene expression levels might be influenced by both cell internal cues (De-Leon and Davidson, 2007; Ralston, 2008) and external cues (Engler et al., 2006; Eyckmans et al., 2012; Hess et al., 2012a; Dingal et al., 2014; Najafabadi et al., 2016). Experimental studies (Mousavi and Hamdy Doweidar, 2015) have shown that the *in vitro* differentiation of hMSC into cells of distinct functional types can be controlled by external factors. Therefore, stem cell differentiation mediated by external factors is a compelling approach that has led to the development of bio-implants, for clinical applications in regenerative medicine.

The applied electric field (EF) is one of the proven external factors known to influence hMSCs dynamics such as migration (Ciombor and Aaron, 1993; Schemitsch and Kuzyk, 2009; Banks et al., 2015; Funk, 2015), elongation (Rajnicek et al., 2008; Tandon et al., 2009), proliferation (Hartig et al., 2000; Lohmann et al., 2000; Kim et al., 2009; Sun et al., 2009), and differentiation (Jansen et al., 2010; Hess et al., 2012b; Petecchia et al., 2015; Miyamoto et al., 2019; Rohde et al., 2019). Comparing these studies, it is evident that the results are inconsistent and show the disparity. While several works have demonstrated an increase in proliferation after exposing cells to EF or electromagnetic field (EMF) (Hartig et al., 2000; Chang et al., 2004; Kim et al., 2009; Sun et al., 2009), others did not detect significant differences or had recorded reduced cell number following EMF exposure (Lohmann et al., 2000; Schwartz et al., 2008; Jansen et al., 2010). Similarly, stimulation effects on osteogenic differentiation are also controversial, ranging from no effects (Chang et al., 2004; Lin and Lin, 2011) to a high increase in the expression of bone-related gene markers (Hartig et al., 2000; Schwartz et al., 2008; Jansen et al., 2010). Due to the complex parameters and the different experimental approaches used, it is difficult to compare these results among each other. In addition, the choice of stimulation method can also influence cellular behavior.

These methods consist of direct or indirect electrical stimulation of the tissue (Schemitsch and Kuzyk, 2009). In the direct stimulation method, the electrodes are placed in contact with the targeted tissue. Some of the disadvantages of direct stimulation are the damage caused to tissues by invasive electrodes and the corrosion of the electrodes due to electrochemical processes (Ciombor and Aaron, 1993). The indirect stimulation method includes capacitive coupling and inductive coupling of electromagnetic fields (EMF). The capacitive coupling is slightly invasive and provides electrical stimulation to the tissue, whereas non-invasive inductive coupling involves both magnetic and electrical stimulation.

To study the stand-alone effects of the EF on the biological tissue, an *in vitro* setup, which is non-invasive and free from the magnetic fields, is necessary. In this context, Hess et al., have developed a novel *in vitro* transformer-like coupling (TC) setup (Hess et al., 2012a). This approach enables a non-invasive electrical stimulation of *in-vitro* culture of hMSCs with homogeneous EF in the cell culture chamber. The TC setup exerts pure EFs to the cell culture, with negligible magnetic field strength (see section 2.1). Thus allowing direct correlation of observed results solely to EF stimulation.

Besides the experimental evaluations, there is a great interest in mathematical modeling and simulation to (i) further gather an in-depth understanding of the cellular mechanism underlying the stem cell response to EMFs, and (ii) to predict optimal stimulation parameters. Fricke (1953) was the first to introduce an empirical equation for the electric potential induced in an ellipsoidal cell in suspension when exposed to an external EF. The first theoretical description (analytical solution of Laplace equation) for the induced potential in a spherical cell in suspension exposed to external EF was given by Schwan (1994) where a spherical shell representing the membrane approximates the cell. This Schwan model treats the cell as a non-conducting membrane subjected to both constant and alternating external EF (Grosse and Schwan, 1992). Schwan's theory has been extended by Kotnik et al. (1997) by considering the conductivity using constant, oscillating, and pulsed EF. Later other geometries such as cylindrical, spheroidal, and ellipsoidal cells suspended in the medium were investigated (Gimsa and Wachner, 2001a,b; Valic et al., 2003; Maswiwat et al., 2008). To determine the induced EF in the internal membranes of the cells, the cells were modeled as multiple concentric shells (Kotnik and Miklavčič, 2006; Vajrala et al., 2008). Several techniques were also employed to examine different cells of complex shapes suspended in an electrolyte, for example, Finite Element Models (FEM) (Miller and Henriquez, 1988; Sebastián et al., 2004; Meny et al., 2007; Ying and Henriquez, 2007), Transport Lattice Models (TLM) (Gowrishankar and Weaver, 2003; Stewart et al., 2004; Gowrishankar et al., 2013) and equivalent circuit models (Ramos et al., 2003; Schoenbach et al., 2004). The effect of surface charge and membrane conductivity was studied on the induced potential in spherical and non-spherical cell geometries by Kotnik and Miklavčič (2006) and Mezeme and Brosseau (2010).

In little over a decade, the theoretical approaches to study stem cell dynamics have begun (Tabatabai et al., 2011; Pisu et al., 2012; Paździorek, 2014; Sun and Komarova, 2015). Although experiments have shown that the external EF affects cellular processes, the theoretical approaches have mainly focused on the collective dynamics of stem cells (Tonge et al., 2010; Lei et al., 2014; MacArthur, 2014; Paździorek, 2014; Renardy et al., 2018; Farooqi et al., 2019; Sarkar et al., 2019). Such approaches consider the stem cell population as a compartment (Tabatabai et al., 2011; Sun and Komarova, 2012, 2015; Yang et al., 2015) and do not resolve the dependency of processes of individual cells on the external factors (Pisu et al., 2012). To the best of our knowledge, the existing mathematical models have not incorporated the cellular responses of interaction with EF distribution in the cell compartments (Pisu et al., 2012).

In this context, we investigate the influence of applied EFs on the dynamics of an *in vitro* culture of hMSCs in a TC setup (see sections 2.2, 2.3). Our mean-field theoretical framework takes into account processes at the scale of an individual stem cell and describes the dynamics of a stem cell population (see sections 3.1 and 3.2). We compare our theory with experimental results reported by Hess et al., and provide a quantitative explanation for the observed behavior of the total number of cells and the total alkaline phosphatase (ALP) activity over time.

## 2. Previous Experimental Results

Our data-driven modeling is based on previous experiments by Hess et al. We use the time dependent experimental data from Hess et al., to study the effect of EFs on hMSC proliferation and differentiation. In the following subsections we recapitulate the experimental TC setup and quantification procedure introduced in Hess et al. We then discuss the corresponding experimental results of the total number of cells and the total ALP activity in the stimulation chamber, which forms the basis for our general theoretical framework.

### 2.1. Electrical Stimulation With TC-Induced Electrical Field

The hMSCs were isolated from bone marrow aspirates of 3 healthy male donors between the age of 20 to 40 years old (for more details on isolation and expansion of cells in Hess et al., 2012a). In a spinner flask containing expansion medium (exm, Dulbecco's modified Eagle's medium with 10% fetal bovine serum and 100 I.U./mL penicillin-streptomycin), about 50,000 hMSCs were seeded on a collagen-coated polycaprolactone (PCL) disc-shaped scaffold at 37 °C with 7% CO_2_ for 24 h (Hess et al., 2012a).

**Figure 1:**
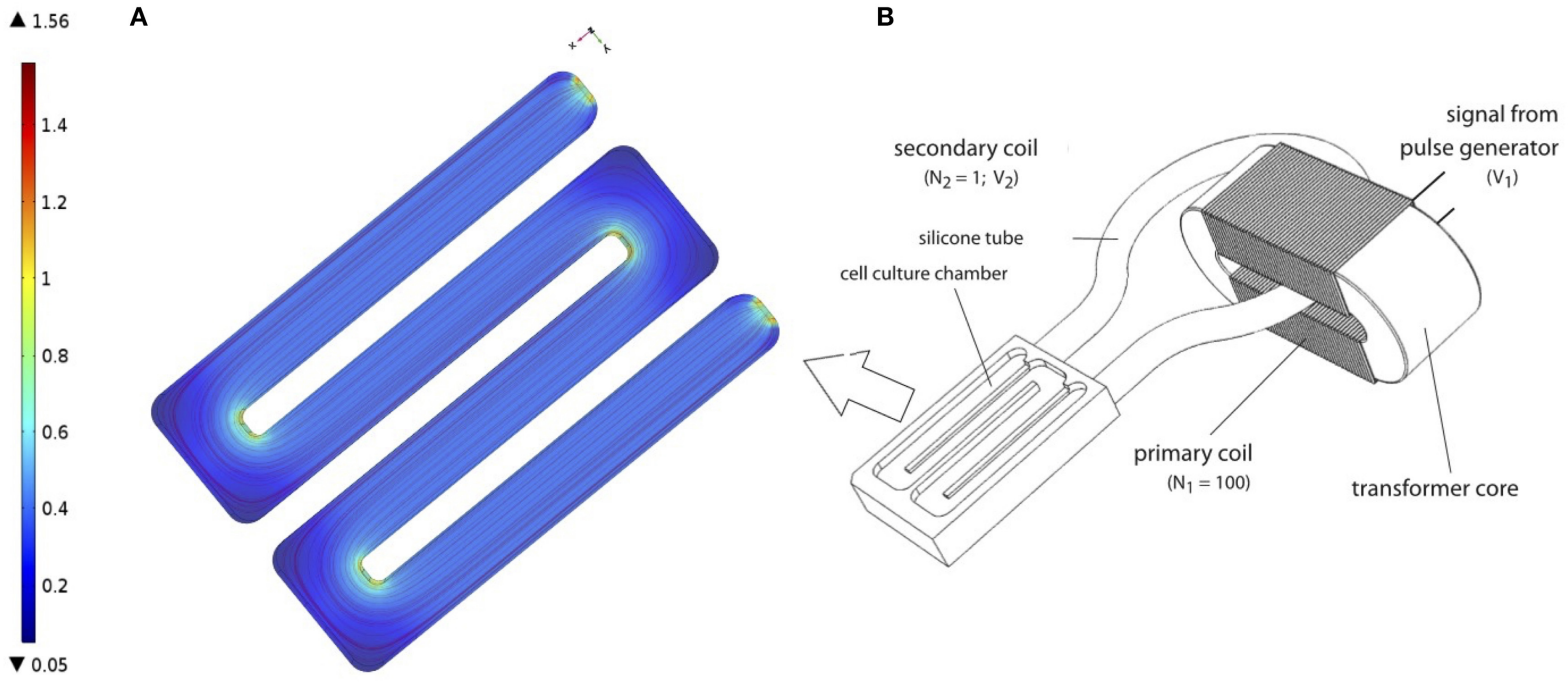
**(A)** Finite Element Model (FEM) of cell culture chamber with the electrical field as described in Hess et al. (2012a). FEM model was performed using COMSOL Multiphysics 4.2a^®^ **(B)** Schematic representation of TC, as described in Hess et al. (2012a). Figure obtained with permission from Hess et al. (2012a).

Subsequently, the PCL-scaffolds with hMSCs were transferred to a cultivation chamber of the transformer-like coupling (TC) system previously described (Hess et al., 2012a) and prepared for electrical stimulation (Figure 1B). In each cultivation chamber, only PCL-scaffolds seeded with the same donor were allowed, so as to avoid side-effects induced by endocrine signaling between hMSC from different donors. Next, 100 ml osteogenic differentiation medium (osm) composed of exm supplemented with 10 nM dexamethasone, 0.2 μM ascorbic acid and 10 mM β-glycerophosphate (all Sigma Aldrich); was added to each cultivation chamber and incubated at 37 °C, 7% CO_2_. Further, medium change was performed every 4 days over the entire course of cultivation. An electrical stimulation regime with rectangular pulses (7 ms, 3.6 mV/cm, 10 Hz) was applied intermittently (4 h stimulation, 4 h pause) over 28 days (Hess et al., 2012a,b). To ensure a homogeneous EF for the cell culture, the cells are seeded on the long arms of the chamber where the electrical field was uniform. Our FEM simulation of the chamber confirms the same (see Figure 1A). Corresponding negative controls without electrical stimulation were set up in identical cultivation chambers, but were not connected to the transformer core.

### 2.2. Total Number of Cells and Total ALP Activity in the TC Apparatus

To study the influence of EFs on stem cell dynamics, cell proliferation and cell differentiation were quantified using standard colorimetric measurement protocol. hMSC proliferation and differentiation were determined via lactate dehydrogenase (LDH) and ALP assay, respectively. Experimental data was recorded on 7, 14, 21, and 28 d after the electrical stimulation regime was applied. Four samples from each condition (control and electrically stimulated) were collected and stored in -80 °C for analyses later as a whole. To prepare the samples for analysis, they were thawed on ice for 30 min, followed by cell lysis for 50 min in cold lysis buffer consist of 1% w/v Triton X-100 / Phosphate buffer saline (PBS). To determine ALP activity at each time point, 25 μl cell lysate was added to 125 μl ALP substrate consisting 1 mg/ml p-nitorphenyl phosphate (Sigma Aldrich), 0.1 M diethanolamine, 1 mM MgCl_2_ and 0.1% w/v Triton X-100/PBS, pH 9.8. The reaction was prepared in 96-well microplate, incubated at 37 °C for 30 min and stopped with 73 μl NaOH. This was followed by centrifugation at 16,000 g for 10 min and 170 μl of supernatant from individual well was transferred to a new 96-well microplate. The absorbance was measured on TECAN microplate reader at 405 nm and corresponding negative controls had used lysis buffer instead of cell lysate. ALP activity was interpreted as μmol para-nitrophenol (pNP) per 10^6^ cells. To determine the cell number present in each scaffold over time, 50 μl of cell lysate was added to equal volume of LDH substrate (Takara, France) in a 96-well microplate and incubated at room temperature for 5 min. The reaction was stopped by adding 50 μl 0.5 M HCl to each well and the absorbance was measured on TECAN microplate reader at 492 nm. The cell numbers were determined by correlating the measured values against a calibration curve derived with defined number of hMSCs. For both assays, the measurements were done in triplicates to increase the accuracy.

### 2.3. Experimental Results

**Figure 2:**
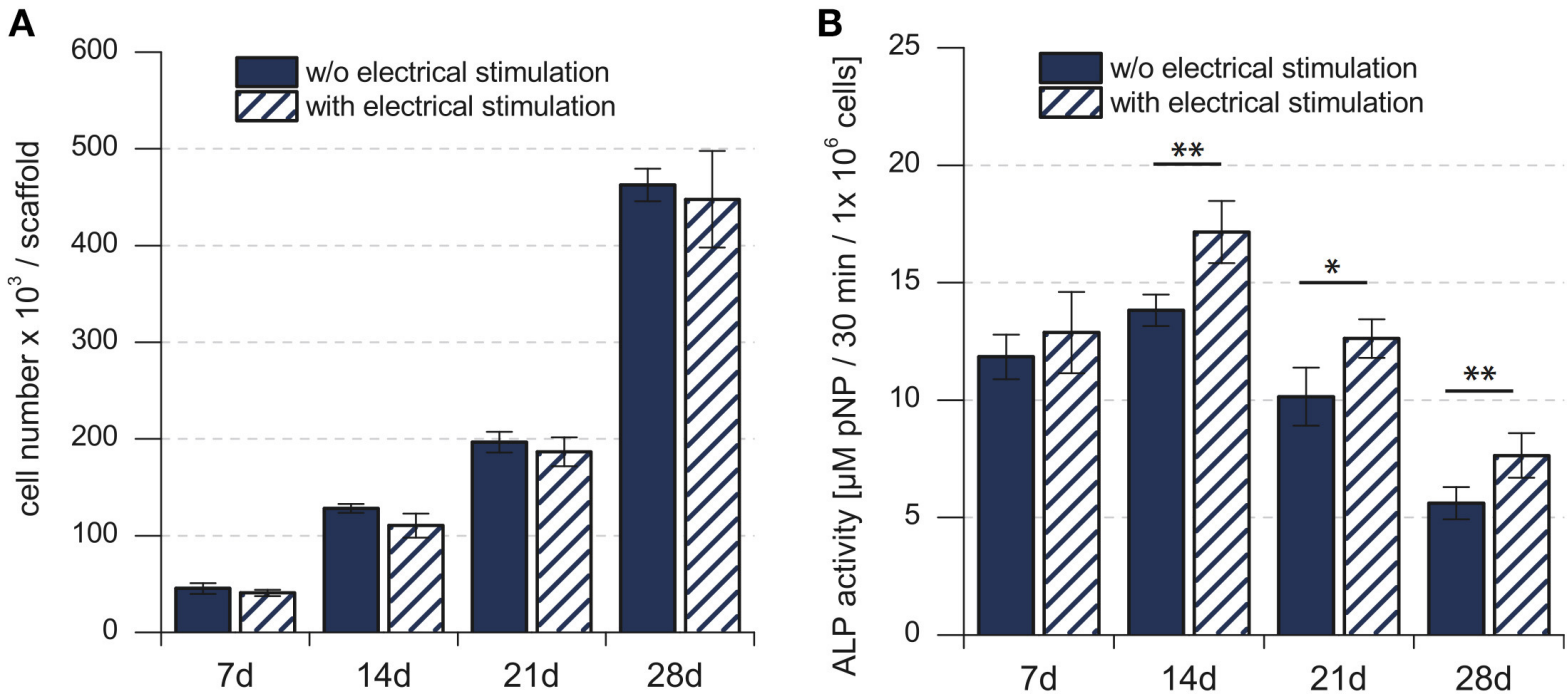
**(A)** Total number of mesenchymal stem cells in the cell culture. **(B)** Biochemical analysis of the total ALP activity in the cell culture. Total number of hMSCs indicated stem cell proliferation, whereas the total ALP activity indicated the stem cell differentiation. ^*^p < 0.05, ^**^p < 0.01. Figure obtained with permission from Hess et al. (2012a).

Cell proliferation, indicated by the change in the total number of cells over time, showed continuous increase for 28 days, in both the electrically stimulated samples and the non-stimulated control samples (Figure 2A). Statistical analysis showed no detectable differences in the total number of cells between stimulated and non-stimulated samples (Hess et al., 2012a). The total ALP activity increased over time and reached the peak after 14 days, followed by a decrease until 28 days. The statistical analysis showed significant difference between the electrically stimulated and non-stimulated control samples. The ALP activity in the electrically stimulated samples was 30% higher than the non-stimulated control samples. This indicated a role of applied EFs in the differentiation process.

## 3. Mathematical Modeling

Based on the experimental data, discussed in the previous section, we made the following observations. First, the non-stimulated control samples show different time-dependent behaviors for the total number of cells and the total ALP activity. Second, the applied EF significantly influences only the time-dependent behavior of the total ALP activity. In order to provide a quantitative explanation for these observations, we formulated a general theoretical description of stem cell dynamics.

### 3.1. General Theoretical Framework for Stem Cell Dynamics

**Figure 3:**
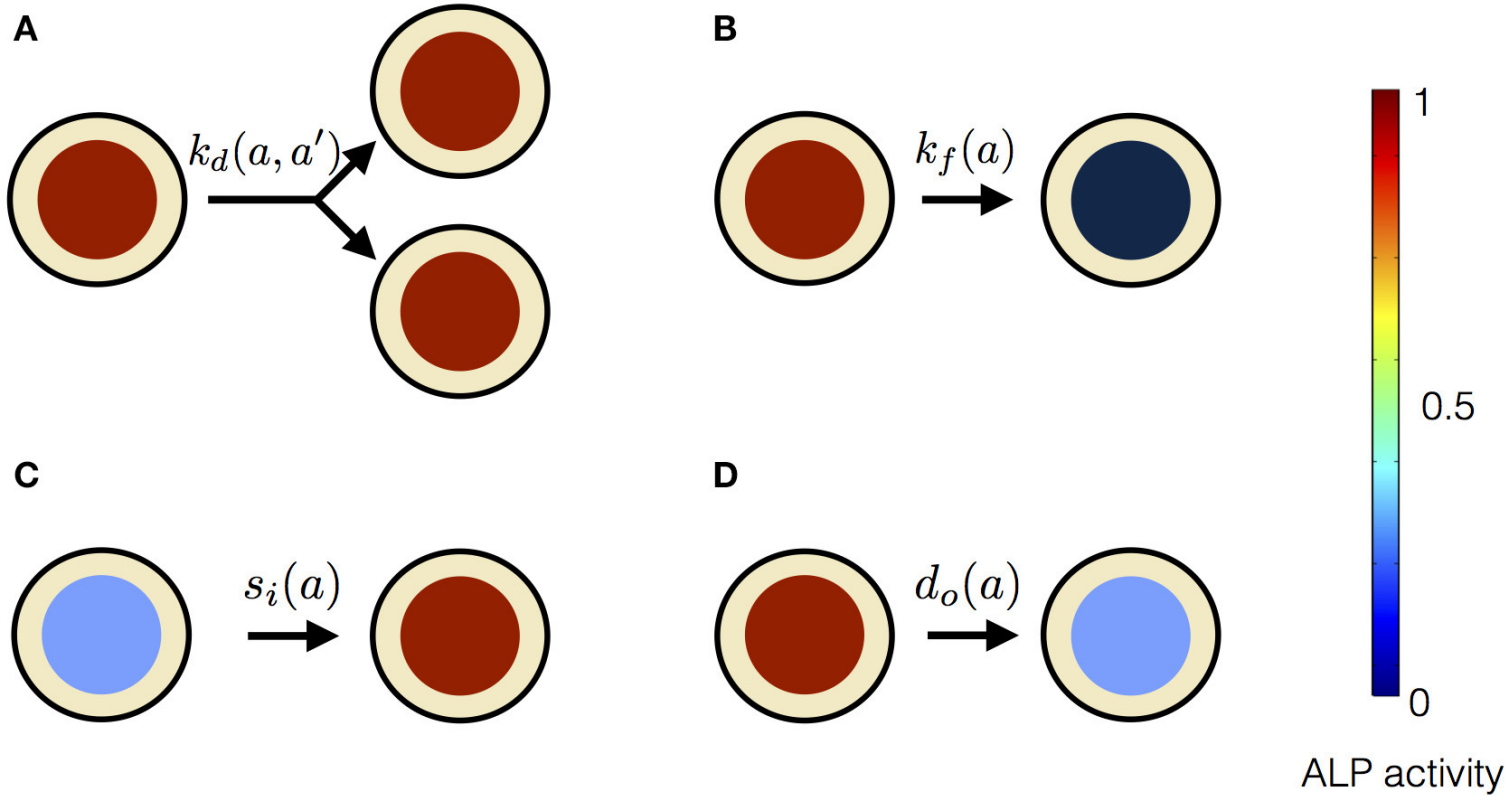
Processes affecting the distribution of ALP activity *n*(*a, t*) in the population of hMSCs. **(A)** When a cell with ALP activity *a* + *a*′ divides, it is replaced by two new cells with ALP activity *a* and *a*′. The colored circles represent the level of the ALP activity of the cell, according to the color scale. High level of the ALP activity is represented by red color, whereas no ALP activity is represented by black color. Such divisions occur at the rate $k_{d}\left(a,a^{\prime }\right)$ and result in the stem cell proliferation. **(B)** A cell can instantaneously lose detectable ALP activity because of terminal osteogenic differentiation into an osteoblast. Such processes occur at the rate *k*_*f*_(*a*). **(C)** ALP activity in a cell can increase due to intracellular ALP synthesis, denoted by influx *s*_*i*_(*a*). **(D)** ALP activity in a cell can decrease due to intracellular ALP degradation, denoted by out-flux *d*_*o*_(*a*).

In our mean-field model, the time-dependent behavior of the stem cell population augments from the processes at the scale of a single stem cell (Figure 3). An individual stem cell undergoes division, giving rise to new cells and thus sustaining the stem cell population. The ALP activity of a stem cell is maintained by the intracellular biochemical processes. Subsequently, a stem cell leaves the stem cell population due to terminal differentiation. Taking these processes into account at the scale of an individual cell, we describe the state of the mesenchymal stem cell population by *n*(*a, t*), where *a* is the ALP activity of a cell in the stem cell population at time *t*. Precisely, *n*(*a, t*)Δ*a* is the total number of cells with ALP activity in the range *a* and *a* + Δ*a* at the time *t*. Generally, *n* can depend on multiple variables besides intracellular ALP activity, such as the cell size, the ALP gene expression of the cell, the EF strength experienced by the cell, the orientation of the cell with respect to the applied EF etc. The change in *n* over time reflects the dynamics of individual stem cells.

(1) $$\begin{array}{l} \frac{\partial n\left(a,t\right)}{\partial t}=-\frac{1}{2}\int_{0}^{a}n\left(a,t\right)k_{d}\left(a-a^{\prime },a^{\prime }\right)da^{\prime }\\ \;+\int_{0}^{\infty }n\left(a+a^{\prime },t\right)k_{d}\left(a,a^{\prime }\right)da^{\prime }-\frac{\partial \left(n\left(a,t\right)s_{i}\left(a\right)\right)}{\partial a}\\ \;+\frac{\partial \left(n\left(a,t\right)d_{o}\left(a\right)\right)}{\partial a}-k_{f}\left(a\right)n\left(a,t\right)\;. \end{array}$$

Equation (1) is based on the Smoluchowski equation describing coagulation phenomena (Smoluchowski, 1916). Similar equations have been studied in a variety of other problems (Baskaran and Marchetti, 2008; Foret et al., 2012; Lade et al., 2015). The first two terms on the right hand side of Equation (1) represent cell divisions in the stem cell population. These cell divisions result in stem cell proliferation. Such cell divisions occur at the rate $k_{d}\left(a,a^{\prime }\right)$, and replace a cell having ALP activity *a* + *a*′, with two daughter cells having ALP activities *a* and *a*′, respectively. These cell divisions described in Equation (1) conserve the ALP activity. In the case of non-conserved cell divisions, both daughter cells have the same measure of ALP activity as the dividing parent cell. Such cell divisions will be described by only one term, namely, $\int_{0}^{\infty }da^{\prime }n\left(a,t\right)k_{d}\left(a,a^{\prime }\right)$ instead of two terms as in Equation (1). The third and the fourth term on the right hand side of Equation (1) represent the flux of ALP activity in the cell due to the intracellular biosynthesis and degradation of ALP, respectively. In our theoretical description we assume the ALP activity of a stem cell to be regulated by the intracellular ALP biosynthesis and degradation processes. For a cell with ALP activity *a*, *s*_*i*_(*a*) is the average ALP activity gained per unit time due to ALP biosynthesis and *d*_*o*_(*a*) is the average ALP activity lost per unit time due to ALP degradation. The last term on the right hand side of Equation (1) represents the loss of a cell with ALP activity *a* from the population. Such losses occur at the rate *k*_*f*_(*a*) due to instantaneous differentiation of a hMSC into a fully differentiated osteoblast cell. Our theoretical framework describes the dynamics of a population of undifferentiated hMSCs, and does not include osteoblasts, i.e., terminally differentiated hMSCs. In our description, cells with measurable ALP activity are classified as undifferentiated mesenchymal stem cells. We assume the osteoblasts to have lower ALP activity, compared to the undifferentiated mesenchymal stem cells. We also assume that the intracellular ALP activity reaches its maximum in the mesenchymal stem cells undergoing differentiation. Now, we introduce two quantities *N*(*t*) and Φ(*t*) as follows,

(2) $$\begin{array}{l} N\left(t\right)=\int_{0}^{\infty }n\left(a,t\right)da,\;\Phi \left(t\right)=\int_{0}^{\infty }an\left(a,t\right)da, \end{array}$$

where, *N*(*t*) represents the total number of cells, and Φ(*t*) represents the total ALP activity in the hMSC population at time *t*. The time-dependent behavior of *N* and Φ describe the dynamics of hMSC population as whole. Using Equation (2) and Equation (1), we can write down the balance relations for *N* and Φ, in the case of cell divisions that conserve ALP activity, as follows,

(3) $$\begin{array}{l} \frac{dN}{dt}=\frac{1}{2}\int_{0}^{\infty }\int_{0}^{\infty }k_{d}\left(a,a^{\prime }\right)n\left(a+a^{\prime },t\right)dada^{\prime }\\ \;-\int_{0}^{\infty }k_{f}\left(a\right)n\left(a,t\right)da\; \end{array}$$

(4) $$\begin{array}{l} \frac{d\Phi }{dt}=\int_{0}^{\infty }s_{i}\left(a\right)n\left(a,t\right)da-\int_{0}^{\infty }d_{o}\left(a\right)n\left(a,t\right)da\;\\ \;-\int_{0}^{\infty }ak_{f}\left(a\right)n\left(a,t\right)da. \end{array}$$

From Equations (3) and (4) we see that the macroscopic quantities *N* and Φ result from the dynamics of individual cells, such as cell division, ALP biosynthesis and degradation governing cellular ALP activity and cell differentiation. The details of the calculation involved in the derivation of Equations (3) and (4) are presented in the Supplementary Material (section 2).

## 4. Results

The experimentally observed time-dependent behavior of *N* and Φ can be explained by a model that includes stem cell proliferation due to cell divisions and osteogenic differentiation. Mesenchymal stem cell differentiation into an osteoblast could either occur instantaneously or proceed gradually giving rise to intermediate pre-osteoblasts with detectable ALP activity. Our theoretical framework distinguishes between these two subtly different processes, which will be discussed in the following.

### 4.1. Progressive Stem Cell Differentiation Model

**Figure 4:**
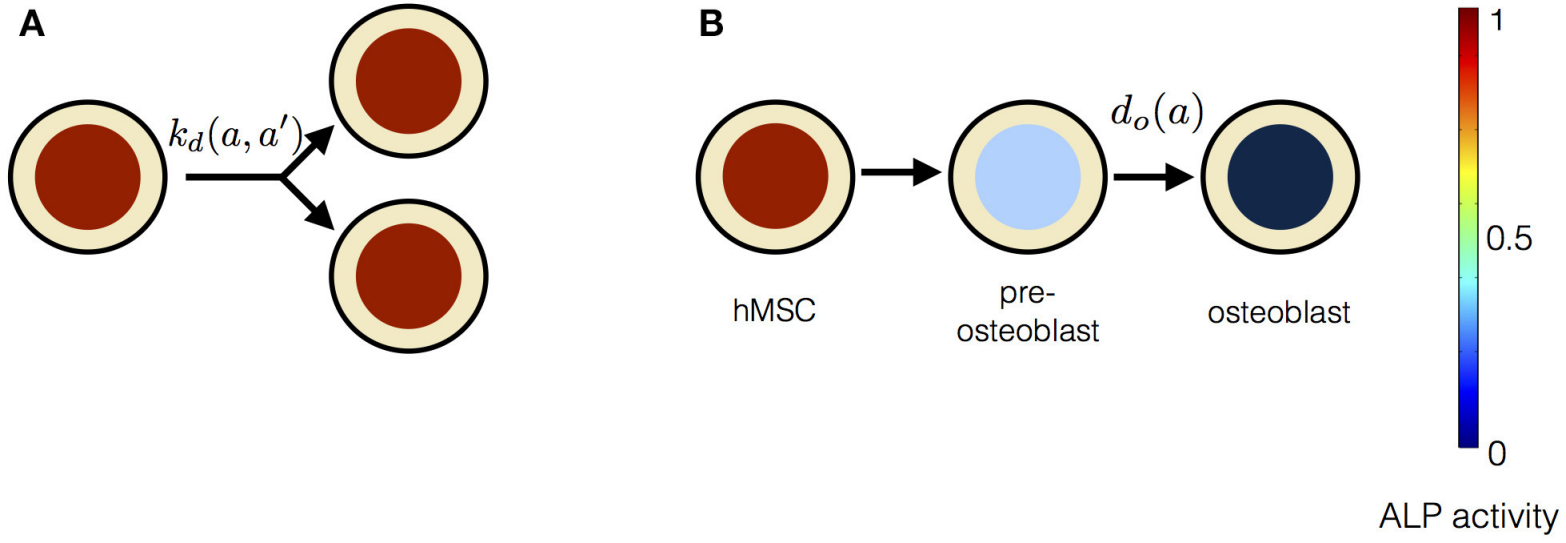
The constituents of progressive stem cell differentiation model are **(A)** Symmetric non-conserved cell division. The division of a stem cell with ALP activity *a* results in two daughter stem cells, each with ALP activity *a*. **(B)** Osteogenic differentiation. The osteogenic differentiation in Model 1 proceeds via gradual loss of ALP activity. Such a process gives rise to temporary states of intermediate pre-osteoblasts.

In this model, stem cell proliferation occurs due to symmetric non-conserved cell divisions. The osteogenic differentiation in this model is due to a gradual decrease in ALP activity via intracellular ALP degradation. The parameter choice for this model is given in Table 1. The cell division in this model occurs at the rate of *k*_*d*_ and results in two daughter cells with the same magnitude of the ALP activity as the parent cell. The stem cell differentiation in this model occurs gradually at the rate of *d*_*o*_, i.e., ALP out-flux due to intracellular ALP degradation is proportional to the cell's ALP activity (Figure 4). The dynamic equation for *n*(*a, t*) using the parameter choice listed in Table 1 is given by,

(5) $$\begin{array}{l} \frac{\partial n\left(a,t\right)}{\partial t}=k_{d}n\left(a,t\right)\int_{0}^{\infty }\delta \left(a-a^{\prime }\right)da^{\prime }-d_{o}\frac{\partial n\left(a,t\right)}{\partial a}\;. \end{array}$$

Equation (5) is solved by using the Laplace transformation technique (Supplementary Material) to obtain the balance relations for *N* and Φ. The time rate of change of *N* is,

(6) $$\begin{array}{l} \frac{dN}{dt}=k_{d}N\;. \end{array}$$

**Figure 5:**
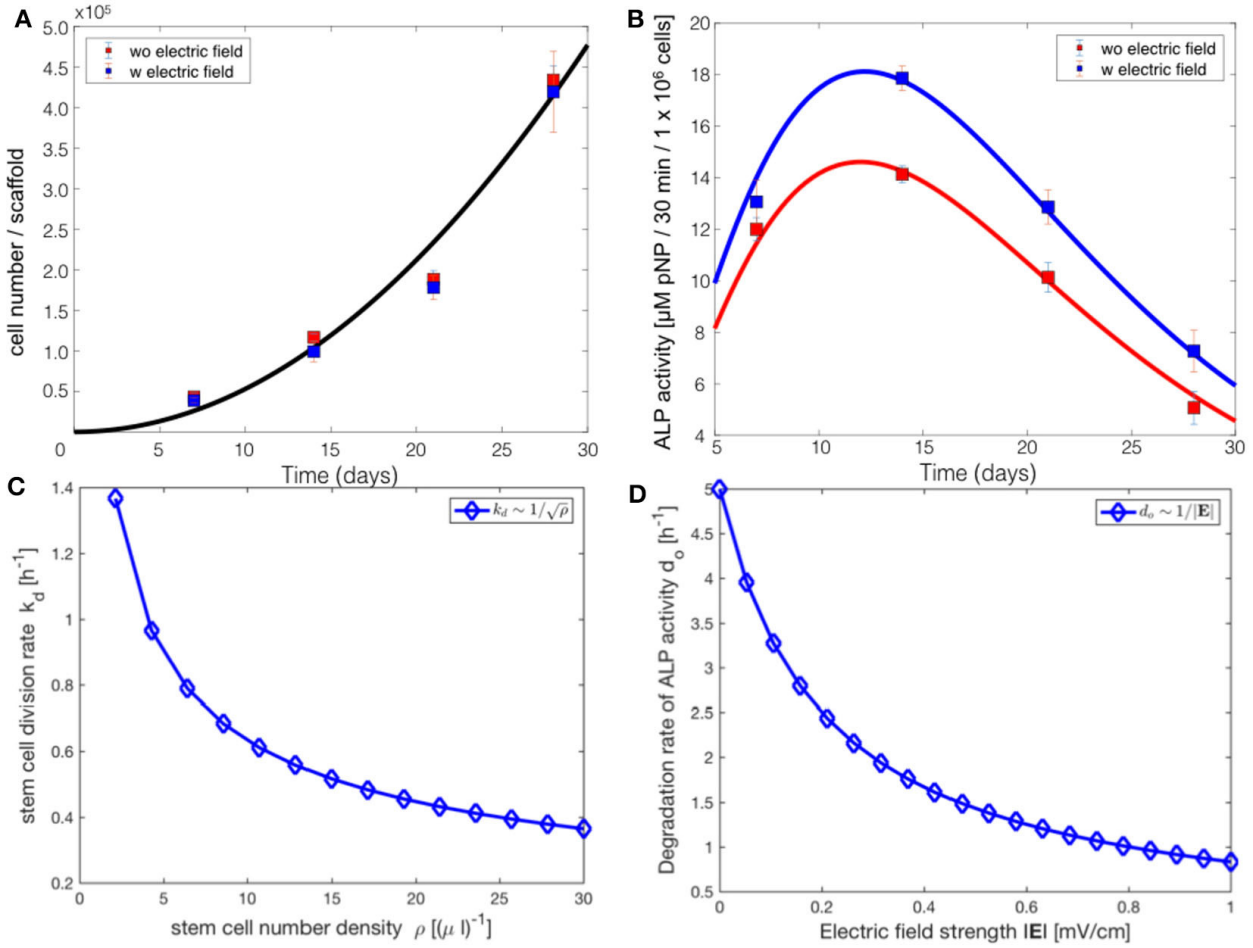
Comparsion of the progressive stem cell differentiation (PSCD) model with experimental results for electrically stimulated (blue box) and non-stimulated (red box) cell culture samples after 7, 14, 21 and 28 days. **(A)** The total number of cells in a scaffold over time. The solid curve is the fit of the analytical solution of PSCD model, given by Equation (7), to the experimental data. **(B)** The total ALP activity of cells in a scaffold cultivated in the stimulation chamber. The solid curve is the fit of the analytical solution of the PSCD model, given by Equation (9), to the experimental data. Error-bars show the standard deviation in the experimental data. **(C)** The result of the fit of the PSCD model to the experimental data for the total number of cells suggests that the divison rate *k*_*d*_ of the stem cells in the hMSC population is inversely proportional to the stem cell number density ρ. **(D)** The result of the fit of the PSCD model to the experimental data for the total ALP activity suggests that the degradation rate of the ALP activity of a cell is inversely proportional to the strength of the applied EF. *d*_*o*_ indicates the rate of osteogenic differentiation.

To fit Equation (6) to the experimental data of the total number of stem cells at each time point in the stimulation chamber, we used *k*_*d*_ = 2/*t*. The solution of Equation (6) with this choice of *k*_*d*_ is given by,

(7) $$\begin{array}{l} N\left(t\right)={\overset{-}{N}}_{0}t^{2}, \end{array}$$

where ${\overset{-}{N}}_{0}=\frac{N_{0}}{t_{0}^{2}}$ and *N*_0_ is the total number of cells showing ALP activity in the hMSC population at the initial time *t*_0_. The fitting was performed using the least squares fitting method, giving an estimate for ${\overset{-}{N}}_{0}=535\pm 40$ (Figure 5A). The χ^2^ value is equal to 36.789 for the fit shown in Figure 5A. The dynamic equation for Φ, obtained from Equation (5), is

(8) $$\begin{array}{l} \frac{d\Phi }{dt}=k_{d}\Phi -d_{o}\Phi \;. \end{array}$$

The solution of Equation (8), with our choice of *k*_*d*_ = 2/*t*, is

(9) $$\begin{array}{l} \Phi \left(t\right)={\overset{-}{\Phi }}_{0}t^{2}e^{-d_{o}t}, \end{array}$$

where ${\overset{-}{\Phi }}_{0}=\frac{\Phi_{0}e^{d_{o}t_{o}}}{t_{0}^{2}}$ and Φ_0_ is the total ALP activity in all the cells in the hMSC population at the initial time *t*_0_. Equation (9) was fit to the experimental data of the total ALP activity at each time point in the stimulation chamber (Figure 5B). Since the statistical analysis showed a significant difference between the non-stimulated control and electrically stimulated samples (Figure 2B), we performed data fitting of each sample separately (Figure 5B). The parameter values of the function, given by Equation (9), obtained as a result of the fit to the experimental data are given in Table 2. The χ^2^ value is equal to 0.972 for the fit shown in Figure 5B to the unstimulated experimental data, and the χ^2^ value is equal to 0.626 for the fit to the electrically stimulated experimental data.

**Table 1:** Choice of parameters for progressive stem cell differentiation model.

*k*_*d*_(*a*, *a*′)	*k*δ_*d*_(*a* − *a*′)
*k*_*f*_(*a*)	0
*s*_*i*_(*a*)	0
*d*_*o*_(*a*)	*d*_*o*_*a*

*This model includes cell proliferation due to symmetric non-conserved cell divisions and gradual differentiation of a hMSC into an osteoblast cell due to out-flux of ALP activity*.

**Table 2:** Parameter values obtained as a result of the fit of Equation (9) to the experimental total ALP activity of stem cells in the stimulation chamber.

	**Control (without ES)**	**With ES**
Φ_0_	0.8007 ± 0.03	0.84 ± 0.034
$d_{o}^{-1}$	5.84 ± 0.09	6.2 ± 0.1

The comparison of the PSCD model with the experimental data suggests that the stem cell division rate decreases with time *k*_*d*_ ~ 1/*t*, whereas the total number of cells grows quadratically *N*(*t*) ~ *t*^2^. Using these two results we analytically derived the dependence of the stem cell division rate on the stem cell density. Our analysis reveals a negative correlation between the divison rate of the stem cells and the stem cell number density, $k_{d}\sim 1/\sqrt{\rho }$ (Figure 5C). The stem cell number density is given by the relation ρ = *N*/*V*, where *N* is the total number of stem cells in the scaffold of the stimuation chamber at time *t*, and *V* is the volume of the scaffold. The volume of the scaffold *V* is fixed. Fitting the PSCD model to the experimental data (see Table 2), reveals an inverse dependence of the degradation rate of the ALP activity *d*_*o*_ on the strength of the applied EF, i.e., *d*_*o*_ ~ 1/|*E*| (Figure 5D).

### 4.2. Instantaneous Stem Cell Differentiation Model

**Figure 6:**
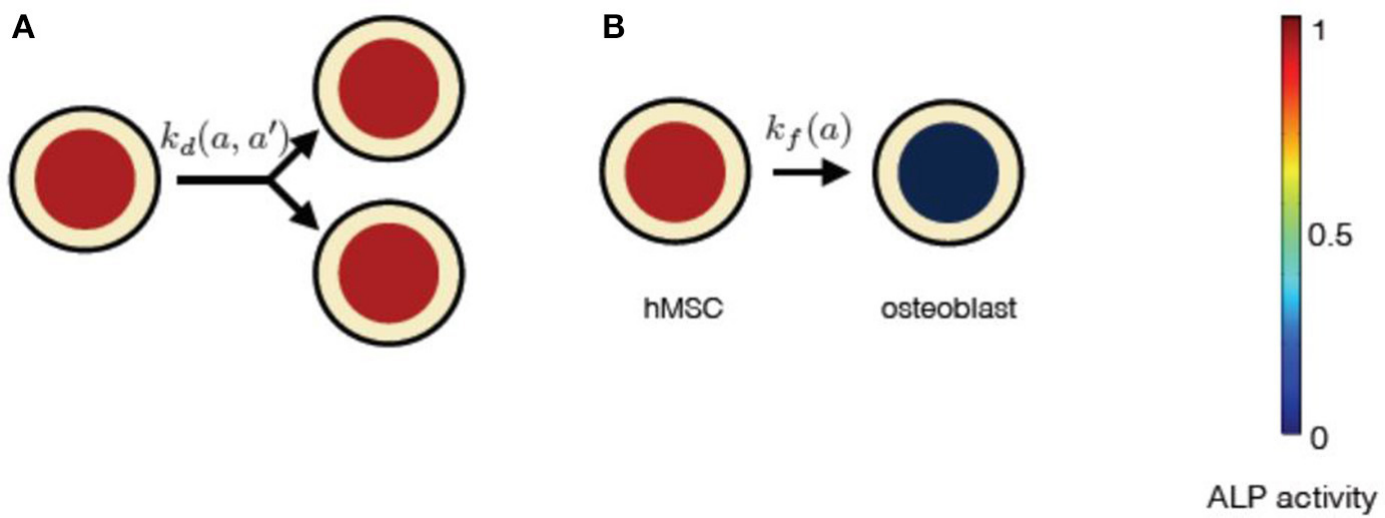
The constituents of progressive stem cell differentiation model are **(A)** Symmetric non-conserved cell division. The division of a stem cell with ALP activity *a* results in two daughter stem cells, each with ALP activity *a*. **(B)**The osteogenic differentiation in instantaneous stem cell differentiation model occurs instantaneously via sudden loss of a cell's ALP activity. Such a process instantaneously gives rise to an osteoblast cell with no ALP activity.

Instantaneous stem cell differentiation model (ISCD) includes stem cell proliferation due to symmetric non-conserved cell divisions, similar to PSCD model. However, the difference between the two models lies in the precise mechanism of osteogenic differentiation. In ISCD model, the differentiation of a stem cell into an osteoblast cell occurs instantaneously, resulting in the total loss of ALP activity in the differentiated osteoblast cell (Figure 6). In this model, such a sudden loss of a cell with ALP activity might also imply apoptosis. The parameter choice for ISCD model is given in Table 3. The dynamic equation for *n*(*a, t*) using the parameter choice listed in Table 3 is given by,

(10) $$\begin{array}{l} \frac{\partial n\left(a,t\right)}{\partial t}=k_{d}n\left(a,t\right)\int_{0}^{\infty }\delta \left(a-a^{\prime }\right)da^{\prime }-k_{f}n\left(a,t\right)\;. \end{array}$$

The change of *N* and Φ over time is,

(11) $$\begin{array}{l} \frac{dN}{dt}=\overset{-}{k}N, \end{array}$$

and,

(12) $$\begin{array}{l} \frac{d\Phi }{dt}=\overset{-}{k}\Phi , \end{array}$$

respectively, where $\overset{-}{k}=k_{d}-k_{f}$.

Since Equation (11) and Equation (12) have exactly the same form, their solutions also have exactly the same functional form. The solution of Equation (11) and Equation (12), by choosing $\overset{-}{k}=k=2/t$ as in Model 1, we get,

(13) $$\begin{array}{l} N\left(t\right)={\overset{-}{N}}_{0}t^{2}, \end{array}$$

(14) $$\begin{array}{l} \Phi \left(t\right)={\overset{-}{\Phi }}_{0}t^{2}, \end{array}$$

where, ${\overset{-}{N}}_{0}=\frac{N_{0}}{t_{0}^{2}}$ and ${\overset{-}{\Phi }}_{0}=\frac{\Phi_{0}}{t_{0}^{2}}$. *N*_0_ and Φ_0_ are as defined in Model 1. The ISCD model describes the experimental data for the total number of cells, but it fails to capture the non-monotonic time dependent behavior of the experimental data for the total ALP activity. Equation (14) shows a continuous increase of the total ALP activity for all time points, whereas experimental data, for both the control and stimulated samples, show an increase in the total ALP activity only up to 14 days (Figure 2B). After 14 days, the total ALP activity shows a continuous decrease till 28 days in both control and stimulated samples which is not captured by this model (Figure 2B).

**Table 3:** Choice of parameters for instantaneous stem cell differentiation model.

*k*_*d*_(*a*, *a*′)	*k*δ_*d*_(*a* − *a*′)
*k*_*f*_(*a*)	*k*_*f*_
*s*_*i*_(*a*)	0
*d*_*o*_(*a*)	0

*This model includes cell proliferation due to symmetric non-conserved cell divisions and instantaneous differentiation of a hMSC into osteoblast cell*.

## 5. Discussion

In this study we developed a general theoretical framework to describe how applied EFs influence the stem cell dynamics. Our mean-field description of stem cell dynamics augments from elementary processes such as stem cell division, differentiation and intracellular regulation of ALP activity. Current theoretical approaches to the study of stem cell dynamics are based on biochemical assays that consider stem cell population as a whole and do not resolve processes at the scale of individual cells. Although the approaches accounting for the discrete nature of the stem cell population, consisting of many individual cells, are scarce, these do not consider dependencies of the cellular processes on the external EF (Tabatabai et al., 2011; Pisu et al., 2012). Our theoretical framework takes into account processes governing the dynamics of individual cells in the stem cell population. The advantage of our general theory is that it allows for studying the influence of various factors, such as the external EF, on the rates of cellular processes. In addition, our theoretical framework can serve as a useful tool to distinguish between different mechanisms through which cellular processes occur. We tested our theory with *in vitro* electrical stimulation experiments by Hess et al. (2012a). We show that our first model, PSCD model, derived from our general theory, can fully describe the time dependent behaviors of the total number of ALP expressing hMSCs and the total ALP activity in the scaffold cultivated in the stimulation chamber. In this model, stem cell proliferation is due to symmetric non-conserved cell divisions and stem cell differentiation occurs via gradual loss of the ALP activity in the stem cells. The rate of stem cell differentiation in this model depend on the ALP activity of the stem cell. In the second model, referred to as the instantaneous stem cell differentiation model, we studied the cell differentiation due to the sudden loss of ALP activity, and its effect on the stem cell dynamics. The rate at which the stem cell differentiation occur, in this model, is independent of the ALP activity of the stem cell. A comparison of our two precisive models with the experiments suggests that the stem cell differentiation occurs gradually, as described in the progressive stem cell differentiation model. This mechanism of osteogenic differentiation gives rise to pre-osteoblast cells, which confirms the experimental results of Rutkovskiy et al. (2016).

Our analysis reveals a negative correlation between the stem cell proliferation rate and the cell number density. The coupling between the cell proliferation rate and the cell density could either be due to density-dependent inter-cellular signaling or mechanical compression, or both (Eyckmans et al., 2012; Najafabadi et al., 2016). Recent experiments have shown that the *in vitro* osteogenic differentiation is associated with the processing of type-1 collagen and progressive deposition of the extracellular collagen matrix (Hanna et al., 2018). The deposition of the extracellular matrix over time might restrict cells from growing and dividing. This could explain the dependence of cell proliferation rate on the cell density, as our analysis suggests. The density-dependent cell division rate has been explored in other context of cellular systems as well (Hoffmann et al., 2011; Recho et al., 2016).

Our results show that the applied EF influences stem cell differentiation rather than stem cell proliferation, which confirms the experimental result of Hess et al. (2012a). We found that the rate of degradation of the ALP activity is inversely proportional to the applied EF strength. In order to precisely quantify the dependency of the stem cell differentiation on the applied EF, further studies of stimulation of hMSCs with varying field strengths are needed.

Our theoretical framework serves as a first step toward developing a more comprehensive model to study the influence of other electric field parameters, such as mode of electric stimulation (AC or DC), the pulse duration, and the frequency on hMSC proliferation and differentiation. Since our framework includes biological rates that are defined as functions of multiple parameters, it allows for studying the dependence of these rates on various biological and physical factors. This can be done by performing a parametric study that involves extending the functional dependence of the kinetic rates to multiple parameters. Cell migration also plays an important role in tissue regeneration. Our theoretical framework, presented in this study, does not contain spatial information of the cells necessary for studying cell migration. By introducing spatial dimensions into our framework, we will be able to study the influence of electric field parameters on stem cell polarization and, thereby, cell migration.

## 6. Conclusion

We draw the following conclusions from our analysis presented in this study. First, despite the complexity of the process, reflected in the multiplicity in its regulatory steps, we show that the stem cell dynamics can be understood by a simple description that captures vital processes. Secondly, our analysis shows that the applied EFs predominantly influence stem cell differentiation. Thirdly, we show that the progressive stem cell differentiation model thoroughly describes the experimental results of Hess et al. (2012a). This model suggests that the osteogenic differentiation of hMSCs progresses gradually, giving rise to pre-osteoblast cells. Fourthly, our analysis shows that the stem cell division rate is cell density-dependant. Finally, our framework allows us to measure the rates of cellular processes and estimates their dependency on external EF. This method is robust and sensitive since the time dependent macroscopic quantities result from an average over many individual cells and multiple experimental repetitions. This framework could serve as a tool to study the influence of external factors on stem cell dynamics through genetic and chemical perturbation of various cellular processes.

## Data Availability Statement

All datasets generated for this study are included in the article/Supplementary Material.

## Author Contributions

RA and UR have conceptualized the study. RA has designed the project, supervised the work including data analysis, and model development. JD developed the mean-field model and derived analytical results. JD and RA worked on the simple model for dynamics of MSCs. JD performed data fitting. RA has performed the FEM simulations. JD, PL, UR, and RA wrote sections of the manuscript. RA critically revised the manuscript and took the responsibility for the integrity of the study as a whole. All authors contributed to manuscript revision, read, and approved the submitted version.

## Conflict of Interest

The authors declare that the research was conducted in the absence of any commercial or financial relationships that could be construed as a potential conflict of interest.
