# Supplementary material for: A General Theoretical Framework to Study the Influence of Electrical Fields on Mesenchymal Stem Cells
Source: Front Bioeng Biotechnol. 2020 Oct 20;8:557447. doi: 10.3389/fbioe.2020.557447 (PMC7606877; doi:10.3389/fbioe.2020.557447)
Supplement: Supplementary file 1 [file Data_Sheet_1.pdf]

# Supplementary Material

## 1 DERIVATION OF DYNAMIC EQUATIONS FOR $N(T)$ AND $\Phi(T)$

Equations (5) and (10) in the main text are solved using Laplace technique to obtain equations for the total number of cells and the total ALP activity in the stimulation chamber. For the purpose of showing our solution approach, we combine the two equations into one equation, using the parameters choice given in Table (1) and (3), as follows,

$$\frac{\partial n(a,t)}{\partial t} = k_d n(a,t) \int_0^{\infty} \delta(a-a')da' + d_o \frac{\partial n(a,t)}{\partial a} - k_d n(a,t) \quad . \tag{S1}$$

The Laplace transform of $n(a,t)$ is defined as,

$$\tilde{n}(x,t) = \int_0^{\infty} e^{-ax} n(a,t)\, ds \quad . \tag{S2}$$

From Equation (S2) we obtain the following two quantities,

$$N = \tilde{n}(0,t), \tag{S3}$$

$$\Phi = -\frac{\partial \tilde{n}(x,t)}{\partial x}|_{x=0} = -\tilde{n}'(x,t)|_{x_0}, \tag{S4}$$

where, $N$ and $\Phi$ represent the total number of cells and the total ALP activity in these cells at time $t$, as defined in the main text. Using the definition given in Equations (S3) and (S4), the Laplace transformation of Equation (S1) is,

$$\frac{\partial \tilde{n}(x,t)}{\partial t} = k_d \tilde{n}(x,t) - d_o x \left( \frac{\partial \tilde{n}(x,t)}{\partial x} \right) - k_f \tilde{n}(x,t) \quad . \tag{S5}$$

where we have used the initial condition $n(a, t=0) = 0$. Substituting $x = 0$ in Equation (S5), we get, for the total number of ALP expressing cells,

$$\frac{dN}{dt} = k_d N - k_f N \tag{S6}$$

Equation (S6) is the same as Equation (13) in the main text. By substituting $k_f = 0$ in Equation (S6) we recover Equation (8) in the main text. Differentiating Equation (S5) with respect to $x$ we get,

$$\frac{\partial \tilde{n}'(x,t)}{\partial t} = k_d \tilde{n}'(x,t) - d_o \left( \frac{\partial \tilde{n}(x)}{\partial x} \right) - d_o x \left( \frac{\partial^2 \tilde{n}(x)}{\partial x^2} \right) - k_f \tilde{n}'(x,t) \quad . \tag{S7}$$

Insert $x = 0$ in Equation (S5) and using the identity given in Equation (S4), we get, for the total ALP activity in the cell culture chamber,

$$\frac{d\Phi}{dt} = k_d \Phi - d_o \Phi - k_f \Phi \quad . \tag{S8}$$

By substituting $k_f = 0$ in Equation (S8) we recover Equation (10) in the main text. By substituing $d_o = 0$ in Equation (S8) we recover Equation (14) in the main text.

## 2 DERIVATION OF GENERAL BALANCE RELATIONS

In this section the balance relations for the total number of cells and the the total ALP activity given by Equation (3) and (4), respectively, in the main text. We will start at Equation (1) in the main text, given by,

$$\begin{aligned}\frac{\partial n(a,t)}{\partial t} &= -\frac{1}{2}\int_0^a n(a,t)k_d(a-a',a')da' + \int_0^\infty n(a+a',t)k_d(a,a')da' \\ &\quad -\frac{\partial(n(a,t)s_i(a))}{\partial a} + \frac{\partial(n(a,t)d_o(a))}{\partial a} - k_f(a)n(a,t) \quad . \end{aligned} \tag{S9}$$

### 2.1 Balance relation for $N(t)$

Integrating the above equation over $da$ we get,

$$\begin{aligned}\int_0^\infty da\frac{\partial n(a,t)}{\partial t} &= -\frac{1}{2}\int_0^\infty\int_0^a n(a,t)k_d(a-a',a')da'da + \int_0^\infty\int_0^\infty n(a+a',t)k_d(a,a')da'da \\ &\quad -\int_0^\infty da\frac{\partial(n(a,t)s_i(a))}{\partial a} + \int_0^\infty da\frac{\partial(n(a,t)d_o(a))}{\partial a} - \int_0^\infty k_f(a)n(a,t)da \end{aligned} \tag{S10}$$

We know that, according to Equation (2a) in the main text, $N = \int_0^\infty n(a,t)da$. We also know, from the boundary conditions discussed in the main text, that $n(x=0,t) = n(x=\infty,t) = 0$. Using these informations, Equation (S10) simplifies to,

$$\begin{aligned}\frac{dN}{dt} &= -\frac{1}{2}\int_0^\infty\int_0^a n(a,t)k_d(a-a',a')da'da + \int_0^\infty\int_0^\infty n(a+a',t)k_d(a,a')da'da \\ &\quad -\int_0^\infty k_f(a)n(a,t)da. \end{aligned} \tag{S11}$$

The third and the fourth term on the right hand side of Equation (S10) vanish due to the boundary conditions of $n(a,t)$ discussed above and in the main text. We will try to simplify the first term on the right hand side of Equation (S10) as follows.

$$-\frac{1}{2}\int_0^\infty\int_0^a n(a,t)k_d(a-a',a')da'da = -\frac{1}{2}\int_0^\infty\int_0^\infty n(a,t)k_d(a-a',a')\Theta(a-a')da'da \tag{S12}$$

where, we have introduced heaviside step function denoted by $\Theta$ which assumes the value 1 only in the limit $a - a' > 0$, and is strictly 0 outside this limit. $\Theta(a-a')$ streatches the upper limit of the second integrand from $a$ to $\infty$. Next we switch the order of integration and substitute $a - a' = u$ as well change the limit of the integral accordingly as follows,

$$-\frac{1}{2}\int_0^\infty\int_0^\infty n(a,t)k_d(a-a',a')\Theta(a-a')da'da = -\frac{1}{2}\int_0^\infty da'\int_{-a'}^\infty n(u+a',t)k_d(u,a')\Theta(u)du \tag{S13}$$

Since $n(a,t)$ is strictly defined only for $a, a' > 0$ we have,

$$-\frac{1}{2}\int_0^\infty\int_0^\infty n(a,t)k_d(a-a',a')\Theta(a-a')da'da = -\frac{1}{2}\int_0^\infty da'\int_0^\infty n(u+a',t)k_d(u,a')\Theta(u)du \tag{S14}$$

Integrand part of Equation (S14) is the same as the integrand of the second term on the right hand side of Equation (S10) since $u$ is a general variable, just as $a$ or $a'$ defined in the range $0 \leq u < \infty$. Replacing the first term on the right hand side of Equation (S11) with Equation (S14) and simplifying, we get,

$$fracdNdt = \frac{1}{2}\int_0^\infty \int_0^\infty n(a+a',t)k_d(a,a')da'da - \int_0^\infty k_f(a)n(a,t)da. \tag{S15}$$

Equation (S15) is the same as Equation (3) in the main text.

## 2.2 Balance relation for $\Phi(t)$

Integrating the above equation over $ada$ we get,

$$\begin{aligned}\int_0^\infty ada\frac{\partial n(a,t)}{\partial t} &= -\frac{1}{2}\int_0^\infty \int_0^a an(a,t)k_d(a-a',a')da'da + \int_0^\infty \int_0^\infty an(a+a',t)k_d(a,a')da'da \\ &\quad - \int_0^\infty ada\frac{\partial(n(a,t)s_i(a))}{\partial a} + \int_0^\infty ada\frac{\partial(n(a,t)d_o(a))}{\partial a} - \int_0^\infty ak_f(a)n(a,t)da\end{aligned} \tag{S16}$$

According to Equation (2b) in the main text, $\Phi = \int_0^\infty an(a,t)da$. Equation (S16) can re-written as,

$$\begin{aligned}\frac{d\Phi}{dt} &= -\frac{1}{2}\int_0^\infty \int_0^a n(a,t)k_d(a-a',a')da'da + \int_0^\infty \int_0^\infty n(a+a',t)k_d(a,a')da'da \\ &\quad - \int_0^\infty ada\frac{\partial(n(a,t)s_i(a))}{\partial a} + \int_0^\infty ada\frac{\partial(n(a,t)d_o(a))}{\partial a} - \int_0^\infty ak_f(a)n(a,t)da.\end{aligned} \tag{S17}$$

We will try to simplify the first term on the right hand side of Equation (S17) using the same substitutions as discussed in the previous section (derivation of general relation for $N(t)$). The resulting equation is,

$$\begin{aligned}\frac{d\Phi}{dt} &= -\frac{1}{2}\int_0^\infty \int_0^\infty (u+a')n(u+a',t)k_d(u,a')duda' + \int_0^\infty \int_0^\infty n(a+a',t)k_d(a,a')da'da \\ &\quad - \int_0^\infty ada\frac{\partial(n(a,t)s_i(a))}{\partial a} + \int_0^\infty ada\frac{\partial(n(a,t)d_o(a))}{\partial a} - \int_0^\infty ak_f(a)n(a,t)da.\end{aligned} \tag{S18}$$

Since $u$ is a general variable, just as $a$ or $a'$ defined in the range $0 \leq u < \infty$, the first term and the second term on the right hand side of Equation (S18) cancel out. Using the boundary conditions, discussed in the main text as well as in the previous section, and integrating by parts the third term and the fourth term of Equation (S18) we get,

$$\begin{aligned}\frac{d\Phi}{dt} &= \int_0^\infty an(a,t)s_i(a)da - \int_0^\infty an(a,t)d_o(a)da \\ &\quad - \int_0^\infty ak_f(a)n(a,t)da.\end{aligned} \tag{S19}$$

Equation (S19) is the same as Equation (4) in the main text.
